# Putative therapeutic impacts of cardiac CTRP9 in ischaemia/reperfusion injury

**DOI:** 10.1111/jcmm.17355

**Published:** 2022-05-10

**Authors:** Seyyed‐Reza Sadat‐Ebrahimi, Hassan Amini, Reza Rahbarghazi, Paria Habibollahi, Shahrouz Ghaderi, Hadi Rajabi, Aysa Rezabakhsh

**Affiliations:** ^1^ 48432 Cardiovascular Research Center Tabriz University of Medical Sciences Tabriz Iran; ^2^ 48432 Department of General and Vascular Surgery Tabriz University of Medical Sciences Tabriz Iran; ^3^ 48432 Stem Cell Research Center Tabriz University of Medical Sciences Tabriz Iran; ^4^ 48432 Department of Applied Cell Sciences Tabriz University of Medical Sciences Tabriz Iran; ^5^ 48432 Department of Pharmacology and Toxicology Pharmacy Faculty Tabriz University of Medical Sciences Tabriz Iran; ^6^ Institute of Molecular Medicine III Medical Faculty Heinrich‐Heine University Düsseldorf Germany; ^7^ Koç University Research Center for Translational Medicine (KUTTAM) Koç University, School of Medicine Istanbul Turkey; ^8^ 48432 Emergency Medicine & Trauma Care Research Center Tabriz University of Medical Sciences Tabriz Iran

**Keywords:** CTRP9, ischaemia/reperfusion injury, signalling pathways, therapeutic target

## Abstract

Recently, cytokines belonging to C1q/tumour necrosis factor‐related proteins (CTRPs) superfamily have attracted increasing attention due to multiple metabolic functions and desirable anti‐inflammatory effects. These various molecular effectors exhibit key roles upon the onset of cardiovascular diseases, making them novel adipo/cardiokines. This review article aimed to highlight recent findings correlated with therapeutic effects and additional mechanisms specific to the CTRP9, particularly in cardiac ischaemia/reperfusion injury (IRI). Besides, the network of the CTPR9 signalling pathway and its possible relationship with IRI were discussed. Together, the discovery of all involved underlying mechanisms could shed light to alleviate the pathological sequelae after the occurrence of IRI.

## 1 INTRODUCTION

Myocardial infarction (MI), a common ischaemic heart disease, is known as a life‐threatening condition that remains the major cause of mortality globally.^1^ Reperfusion therapy is known as the key strategy in managing MI, resulting in significantly reduced mortality rate, infarct size, and improved left ventricular function. Nevertheless, a serious setback for reperfusion therapy is a process known as ischaemic/reperfusion injury (IRI). This process leads to substantial cell death and has been demonstrated to account for up to half of the final myocardial infarct size.^2^ Despite significant advances in the clinical settings, effective therapies for preventing myocardial IRI remain to be deciphered.^3^ In this regard, evidence points to the suboptimal clinical success of reperfusion strategies in MI patients undergone thrombolytic or primary percutaneous coronary interventions due to IRI, which underscores a requisite of alternative approaches and finding protective mechanisms under these conditions.^4^ It has been shown that several molecular mechanisms and cellular precursors are activated inside the injured cells to display resistance under pathological conditions.^5^ For instance, it was suggested that factors from the C1q tumour necrosis factor‐related proteins (CTRPs) superfamily could be activated during both physiological and pathological conditions. CTRPs, as bioactive cardiokines, are secreted by adipose tissue and cardiac endothelial cells. Further molecular identification of CTRPs family has shown various 15 constituents, including CTRP1 to 15, which are adiponectin (APN) paralogs.^6^ Among CTRPs, CTRP9, with high homology to APN, is one of the well‐established members of CTRPs and appears critical roles in the adult cardiac tissue. Although CTRP9 was initially discovered in adipose tissue, recent studies have shown high levels of CTRP9 in cardiac tissue compared to the other organs.^6^ This fact provides a substantial explanation for the extensive range of CTRP9 metabolic bioactivities during cardiovascular abnormalities, which can be correlated with cardiomyocyte homeostasis. In this respect, the activation of CTPR9 during CVD is an accepted mechanism leading to the inhibition of inflammation, ischaemic/reperfusion injury (IRI) and cardiac remodelling after MI. Likewise, the participation of CTPR9 in glucose homeostasis and endothelium‐dependent vaso‐relaxation has been indicated.^7^

Notably, proteolytic cleavage of full‐length CTRP9 (fCTRP9) in cardiac tissue can contribute to making a globular domain isoform of CTRP9 (gCTRP9). Detailed molecular investigations have indicated that the latter molecule is a highly bioactive isoform and protects the cardiac tissue from the IRI and pathological remodelling after MI.^7^, ^8^ Mechanistically, CTRP9 has a critical function in cell bioactivity through the interaction with the 5’‐adenosine monophosphate‐activated protein kinase (AMPK)‐related signalling pathway in the cardiovascular system. This molecule is considered an effector involved in vascular smooth muscle cell proliferation and relaxation, cholesterol uptake, macrophage phenotype acquisition, cytokine production and plaque stability.^9^, ^10^, ^11^, ^12^ Studies have revealed that IRI can lead to the reduction of plasma and myocardial CTRP9 levels by nearly 50% with an increase of free fatty acid (FFA) levels in plasma.^13^ Whether the activity of CTPR9 under the normal condition can protect the cardiomyocytes against injuries or the reduction of this factor occurs in response to the irreversible condition is a matter of debate. The administration of exogenous CTRP9 can restrict the ischaemic area and reduce the apoptotic cardiomyocytes during experimentally induced MI.^8^, ^13^

Whereas the overexpression of CTRP9 in cardiomyocytes increases the possibility of cardiac dysfunction, also, mice with heterozygous/homozygous CTRP9 knockout represented less cardiac hypertrophy and systolic cardiac function improvement upon pressure overload than the wild types. Such effects may be related to the dose‐dependent activity of CTRP9 in the cardiac tissue. Albeit CTRP9 might have some undesirable effects on cardiac tissue in some conditions, the exact role of CTRP9 in CVD has not been fully deciphered, and further investigations are needed to clarify how CTRP9 affects different signalling pathways during both cardiovascular physiological and pathological events. In this review article, we focused on the recent advances regarding CTRP9 potential therapeutic effects on IRI. How CTRP9, as a target molecule, can modulate specific molecular pathways involved in the restoration of cell function in the cardiac tissue is the main field of query.

## 2 IRI PATHOPHYSIOLOGY

Restoration of the blood flow to the infarcted myocardium is mainly associated with some adverse effects making it a ‘double‐edged sword’. This phenomenon is known as ‘reperfusion injury’ or IRI. Both pre‐clinical and clinical studies have been conducted to determine the underlying mechanisms beyond the multipart pathophysiology of IRI. In addition, reperfusion can exacerbate tissue injury with several mechanisms as addressed below:

### 2.1 Excessive production of oxygen‐free radicals

**FIGURE 1:**
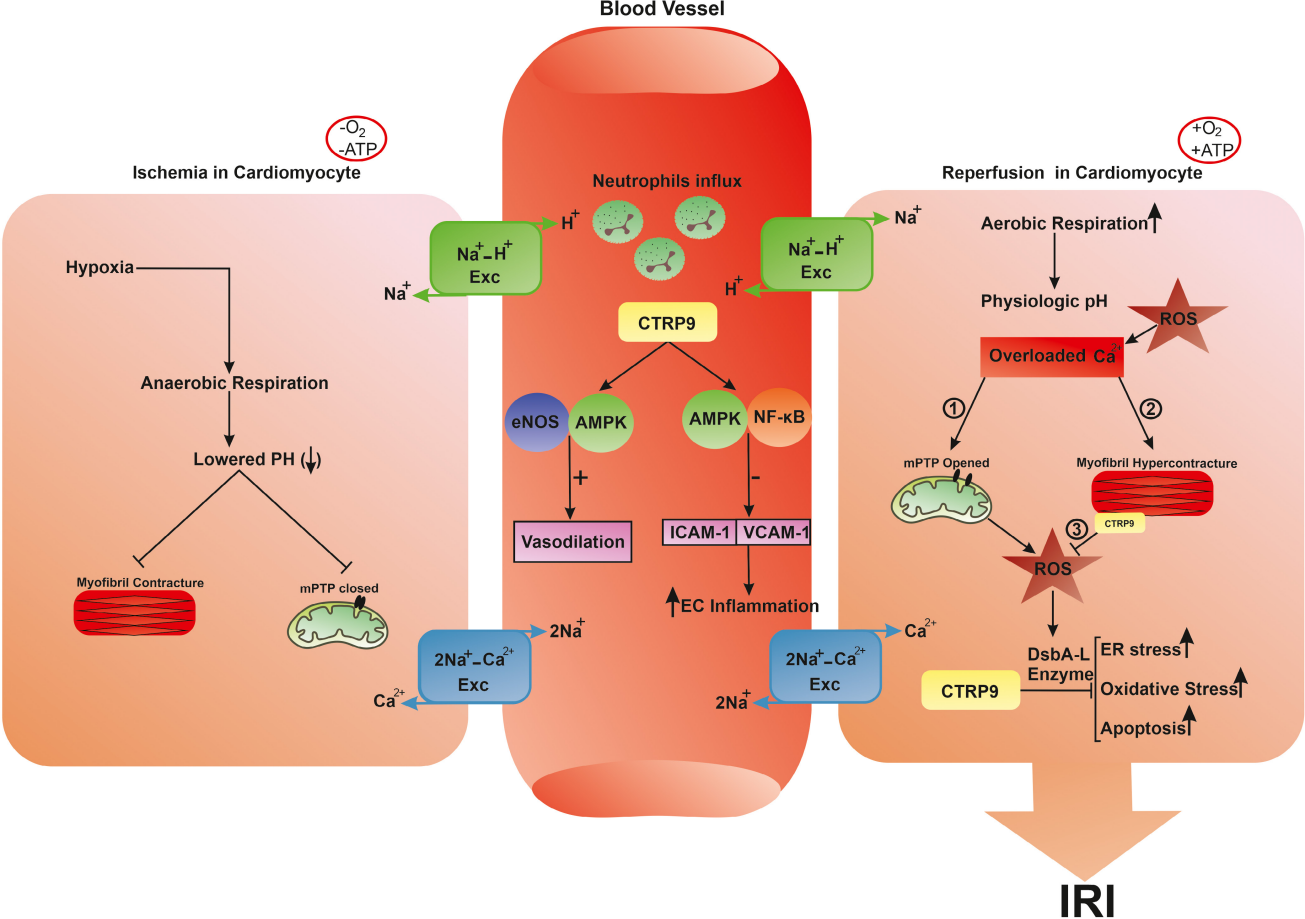
Schematic illustration of IRI pathophysiology in the cardiomyocytes during ischaemic and reperfusion states

This phenomenon mainly occurs in the infarcted tissue when the anaerobic metabolism due to the hypoxic state returns to aerobic metabolism following restoring the oxygen supply by reperfusion of the tissue with fresh blood. However, reintroducing the oxygen promotes reactive oxygen species (ROS) generation. On the contrary, the antioxidant levels in the ischaemic cells remarkably drop due to hypoxic condition and anaerobic metabolism. As a result, the abandoned ROS generation leads to oxidative stress, promoting cell damage, endothelial dysfunction, DNA damage and local inflammatory responses (Figure 1).^14^

### 2.2 Exaggerated inflammatory response

Established oxidative stress and the endothelial cells’ damage trigger an inflammatory response that may lead to a cytokine storm and subsequent tissue damage.^14^ The inflammatory cascade promotes the further release of inflammatory mediators such as interleukins (ILs) and bioactive complements, worsening the myocardial injury.

### 2.3 Electrolyte imbalance

The anaerobic metabolism leads to impairment of the electron transport chain in mitochondria, retention of lactic acid and, finally, dropping ATP production level. Subsequently, decreased level of ATP production due to anaerobic metabolism leads to a failure of Na+‐K+‐ATPase and Ca2+‐ATPase on both cell surface and endoplasmic reticulum. After that, the dysfunction of Na+‐K+‐ ATPase leads to a rise in sodium inside and potassium outside the cells, respectively. Dysregulation of sodium balance impairs the activity of sodium–hydrogen exchanger pumps (Na+‐H+ pumps) and subsequently decreases cellular pH, leading to altered enzyme activity and clumping of nuclear chromatin. Additionally, the dysfunction of calcium pumps on the endoplasmic reticulum site reduces the calcium reuptake. Finally, sodium, calcium and hydrogen retention inside the cells causes cell swelling (Figure 1).^15^, ^16^

### 2.4 Metabolic negative role of free fatty acids

Intriguingly, animal studies have revealed an overshoot in the rate of fatty acid oxidation following myocardial reperfusion. High rates of fatty acid β‐oxidation significantly reduce glucose oxidation.^17^ Furthermore, dissemination of harmful free fatty acids is also demonstrated during myocardial reperfusion, resulting in myocardial uptake and necrosis.^2^

### 2.5 Microembolization and distal plugging

Reperfusion also induces a pro‐thrombotic milieu, facilitating the activation of platelets, namely ‘platelet plugging’ on the microvasculature. Therefore, distal embolization of platelet‐rich thrombus occurs after myocardial reperfusion, leading to diminished myocardial microvasculature perfusion and reducing the clinical benefits of reperfusion therapy, resulting in a phenomenon termed ‘no‐reflow’, happening when patency of the epicardial infarct‐related artery does not bring about the restoration of coronary and microvascular blood flow.^2^

## 3 ADIPO/CARDIOKINES‐RELATED THERAPEUTIC POTENTIALS FOR ISCHAEMIC CARDIOVASCULAR DISEASE

**FIGURE 2:**
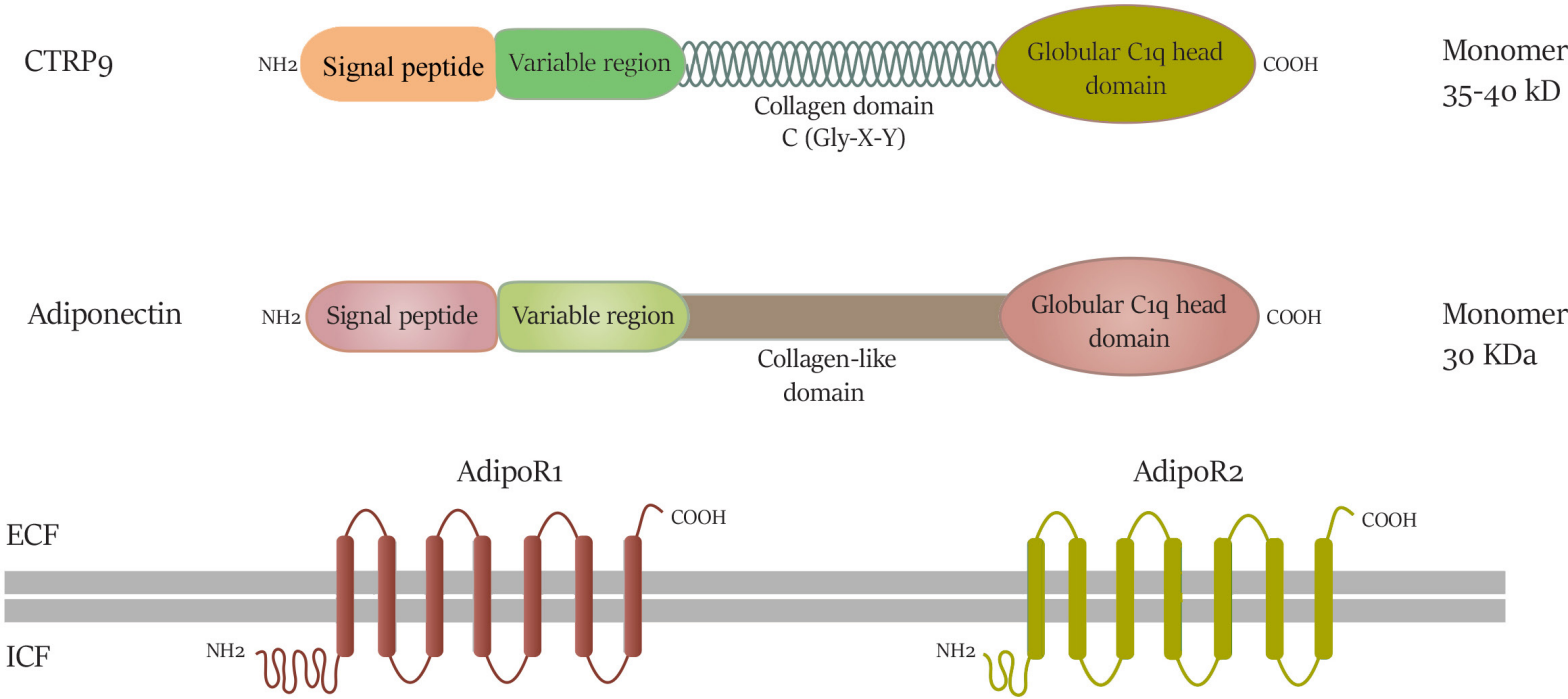
Similar structural characteristics of adiponectin (APN) and CTRP9 molecules

To the best of our knowledge, adipose tissue‐derived APNs are significant substances involved in the haemostasis of metabolic status. Among these, adiponectin is a notable subset of adipokines with well‐known beneficial effects.^18^ Adipose tissue‐derived adiponectin, the most abundant and well‐studied peptide, is considered a therapeutic molecule regarding the multiple effects in metabolic disorders such as obesity and diabetes that serves a dominant protective role in endothelial dysfunction reflecting appreciable anti‐atherogenic, insulin‐sensitizing and anti‐inflammatory effects in both pre‐clinical and clinical settings.^19^ It is also worth noting that some endoplasmic reticulum (ER)‐associated proteins namely ERp44, DsbA‐L, GPR94 and Ero1‐α substantially involve in adiponectin assembly and secretion from adipocytes. The modulatory effects of adiponectin are mainly mediated by adiponectin receptors (AdipoRs), including two isoforms, AdipoR1 and AdipoR2. Beyond the potent metabolic modulatory impacts of adiponectin, according to the pieces of literature, the neuroprotective of this molecule, particularly in cognitive disorders and Alzheimer's disease, has been well‐established.^20^ Given the large structural similarity between adiponectin and CTRP9, as shown in Figure 2, it could assume that there is a plausible functional overlap that drawn great attention to exploring the further therapeutic potential of CTRP9 in terms of cardiometabolic abnormalities.^21^ In this sense, CTRP9 exerts a fine‐tuned anti‐atherogenic effect in type 2 diabetic patients, which can impede the atherosclerosis progression.^22^ Also, due to the established anti‐inflammatory aspect, serum CTRP9, by diminishing inflammatory indicators as well as adjusting lipid profile, is implicated as a promising factor in terms of coronary atherosclerosis treatment in clinical practice.^23^ Besides, CTRP9 following some genetic manipulation exhibited improvement against atrial inflammation, fibrosis and susceptibility to atrial fibrillation in post‐MI periods, in vivo.^24^

### 3.1 Beneficial effects of CTRP9 against oxidative stress in cardiomyocytes

Under physiological conditions, low amounts of ROS are produced in the mitochondrial respiratory transport chain and leak moderately to the cytosol.^25^ With the onset and persistence of ischaemia, the function of the mitochondrial electron chain is altered due to ROS overproduction. Further restoration of oxygen supply by reperfusion exacerbates ROS accumulation and leads to cell toxicity, as well as the antioxidant system overwhelming.^26^ This could be related to direct protein carboxylation, lipid peroxidation and DNA damage.^25^, ^26^ Early studies have also demonstrated a reciprocal relationship between oxidative stress status and CTRP9 production in the ischaemic tissues. The data of the survey conducted by Kambara et al. revealed the inhibitory role of CTRP9 on ROS production in endotoxin‐induced acute cardiac injury model.^8^ The data suggested that myocardial injection of bacterial lipopolysaccharide (LPS) in CTRP9 knockout mice displayed higher levels of ROS compared to the wild‐type mice.^8^ Hence, the loss of CTRP9 allows severe acute cardiac injury upon the LPS injection by the accumulation of ROS. Presumably, CTRP9 in cardiomyocytes could increase the ROS scavenging and clearance via the activation of the antioxidant system.^13^ Even so, direct evidence for CTRP9‐mediated antioxidant capacity or related underlying mechanisms is lacking.

### 3.2 The role endoplasmic reticulum stress in IRI

Endoplasmic reticulum stress (ERS) is characterized by the accumulation of misfolded and dysfunctional proteins inside the ER cisternae. ERS would frequently occur under the stress conditions such as oxidative stress and IRI, ultimately resulting in cell death.^27^ Regarding CTRP9 activity, recent studies have shown that CTRP9 can be involved in the amelioration of IRI‐induced ERS under diabetic conditions. Besides, the moderate activation of chaperones and protein folding procedures in the presence of CTRP9 were reported. CTRP9 can trigger disulfide‐bond‐A oxidoreductase‐like protein (DsbA‐L) enzyme inside the ER, promoting the elimination of misfolded proteins through the lysosomal degradation system.^28^ The overactivity of DsbA‐L can lead to multimerization of adiponectin in the adipose tissue, showing an adiponectin synthesis regulator. Yet, the reciprocal interaction of CTRP9 and DsbA‐L has not been thoroughly addressed. If so, one could postulate that the CTRP9 can reduce cell toxicity by inhibiting the misfolded proteins inside the ER. Despite the existence of evidence for a protective role of CTRP9 against ERS in hepatic cells, further investigations deserve to determine the putative role of CTRP9 on cardiomyocytes after IRI.^28^

### 3.3 Anti‐apoptotic effect of CTRP9 in cardiomyocytes under IRI

**FIGURE 3:**
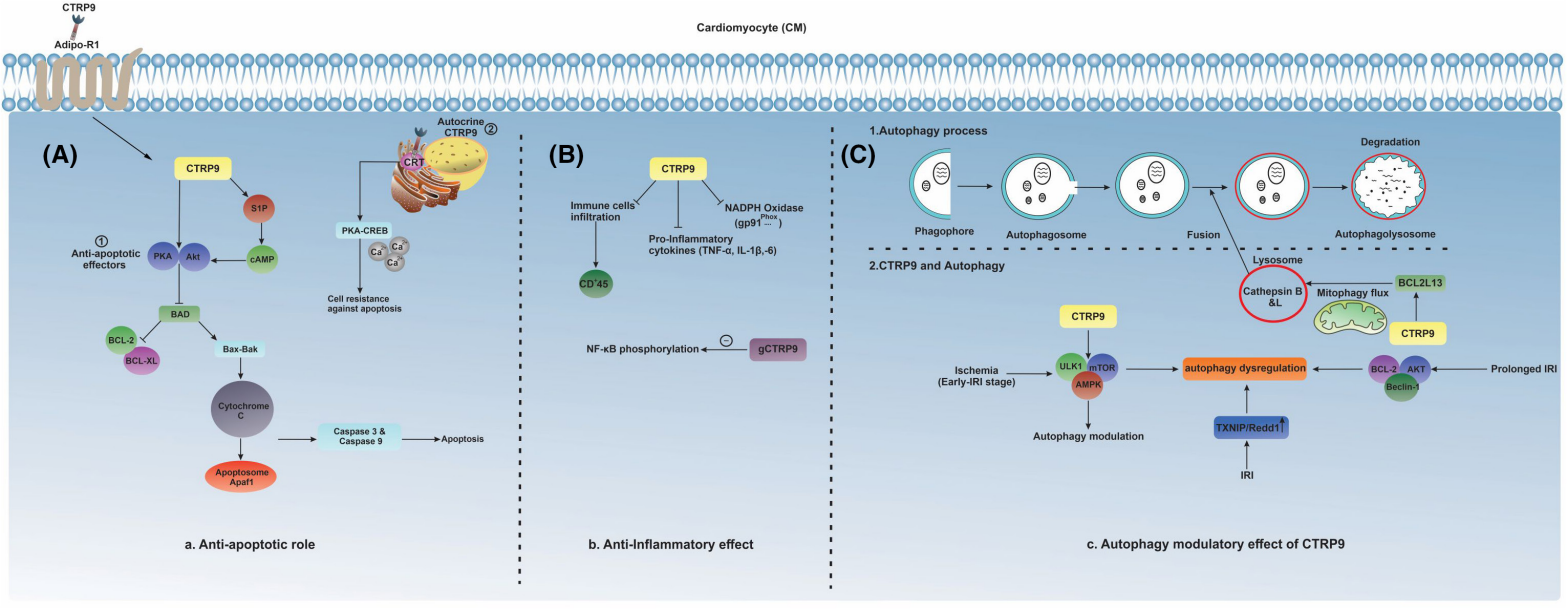
Multiple therapeutic impacts of CTRP9 under IRI. (A) anti‐apoptotic signalling pathway of CTRP9 mediated by AdipoR‐1‐dependent and autocrine manners. AMPK and S1P are two critical regulators of the CTRP9‐related signalling pathway to exert cardioprotective and anti‐apoptotic effects. (B) CTRP9 also represents an autophagy modulatory effect under different stress conditions, for example short and long‐term IRI. (C) anti‐inflammatory effect of CTRP9 by targeting pro‐inflammatory cytokines, NADPH oxidase subtypes and immune cells

Like necrotic changes, apoptotic cell death has been documented after the occurrence of IRI. In this regard, multiple pro‐apoptotic Bcl2 proteins, including Bcl‐2‐associated X protein (Bax), Bcl‐2 homologous antagonist killer (Bak), BH3 interacting‐domain (Bid) and p53 upregulated modulator of apoptosis (PUMA), are upregulated during IRI.^29^ The attachment of these factors to the mitochondrial outer membrane increases the leakage of pro‐apoptotic proteins such as Smac/Diablo, cytochrome C and endonuclease G to the cytosol where the activation of caspase 3 and caspase 9 leads to cell apoptosis via intrinsic apoptosis signalling pathway. Inside the cytosol, Apaf1, an adaptor protein, is connected to cytochrome C, caspase 3 and caspase 9, leading to the activation of apoptosome.^29^ Along with these changes, the suppression of caspase‐inhibitory proteins by Smac/Diablo activity and G endonuclease‐induced DNA destruction can dictate apoptotic changes in the host cells.^29^ Moreover, several lines of evidence have emphasized a prominent anti‐apoptotic role for CTRP9 in IRI through a variety of signalling pathways.^30^ In support of this claim, Kambara et al. indicated that CTRP9 mediates an anti‐apoptotic function mainly through the phosphorylation of AMP‐activated protein kinase (AMPK) (Figure 3a).^13^ The protective role of AMPK during IRI can be obtained by its activity and subcellular distribution.^31^ In parallel, Sun et al. proposed that the crosstalk between the AMPK signalling pathway and CTRP9 leads to the cardioprotective effect. Noteworthy, there is a direct correlation between CTRP9 and AMPK upstream kinase, in particular by protein kinase A (PKA) activity that administration of exogenous CTRP9 can activate both protein kinase B (Akt) and PKA under the ischaemic conditions (Figure 3a).^32^ The activation of BCL2‐associated agonist of cell death (BAD) inhibits anti‐apoptotic B‐cell lymphoma (BCL)‐2 family members (such as BCL‐x and Bcl‐2), coinciding with the promotion of pro‐apoptotic proteins like Bcl‐2‐associated X protein (Bax), and BCL‐2 homologous antagonist killer (Bak) and mitochondrial leakage of cytochrome C. These data suggested the activation of CTRP9 inhibits BAD and relevant downstream effectors following IRI. Another cardioprotective effect of CTRP9 is possibly related to the adiponectin receptor‐1 (adipoR‐1) pathway. This receptor is widely distributed in cardiac tissue, where the attachment of CTRP9 to this receptor can mediate anti‐apoptotic effects on cardiomyocytes after IRI.^13^ Also, Zhao et al. reported a cardioprotective role of CTRP9 in an autocrine manner via binding to the ER‐resident chaperone named calreticulin (CRT) following IRI. The apparent specific activity of CRT relies on calcium concentration. This enzyme strongly binds to misfolded substrates and prohibits trans‐ER‐Golgi transport. N‐link glycosylation and chaperoning of major histocompatibility complex class‐I have the integrity to CRT activity.^30^ Following binding of CTRP9 to CRT, pro‐survival complex PKA‐CREB is activated inside the cardiomyocytes and increases these cells’ resistance.^30^

Attempts to find the anti‐apoptotic effects of other CTRP members showed the participation of CTRP9 in the regulation of apoptosis. Yuasa et al. suggested that the activation of CTRP9 triggers sphingolipid signalling by engaging sphingosine‐1‐phosphate (S1P).^33^ This enzyme is expressed in cardiomyocytes and participates in several biochemical reactions. S1P has an essential function in the elevation of intracellular content of cyclic adenosine 3′, 5′‐monophosphate (cAMP) inside the cardiomyocytes, leading to suppression of apoptotic effectors.^33^ Given the critical role of S1P in apoptosis signalling, pharmacological inhibition of S1P receptors blunts the modulatory effects of CTRP9. It seems that many of the members of the CTRP family are expressed inside the cardiomyocytes in different sites. Whether other members of the CTRP family participate simultaneously or sequentially in the regulation of dynamic growth and apoptosis needs further studies.

### 3.4 Anti‐inflammatory effect of CTRP9 in cardiomyocytes under IRI

The secondary complication caused by IRI has been directly correlated with the production of inflammatory cytokines/chemokines and neutrophil recruitment into the cardiac tissue.^34^ The infiltration of innate immune cells towards the ischaemic tissue is a phenotypic hallmark of IRI early‐stage changes. Among different bioactive molecules produced by recruited immune cells, xanthine oxidase or NADPH oxidase are known to exacerbate oxidative stress and inflammatory responses.^26^ The continuity of inflammation results in IRI induced by autoimmune responses such as natural antibody recognition of neo‐antigens and activation of the complement system.^35^ Upon the activation of immune‐related responses, CTRP9 is recruited to faint the production of pro‐inflammatory cytokines including tumour necrosis factor‐alpha (TNF‐α), interleukin‐1 beta (IL‐1β), IL‐6, monocyte chemoattractant protein 1 (MCP‐1) and NADPH oxidase subsets including gp91^phox^, p67^phox^ and p47^phox^ in the ischaemic tissue.^8^, ^33^ Besides, the expression of TNF‐α and MCP‐1 was inhibited in murine macrophages after treatment with oxidized low‐density lipoprotein (oxLDL).^10^ In this scenario, the activation of CTRP9 would have to govern both its cellular and molecular targets against IRI‐induced inflammation. On the contrary, CTRP9 is involved in suppressing of immune cell infiltration to the site of injury. In line with this report, it has been documented that a high rate of CD45^+^ cell infiltration has been shown in the heart tissue of CTRP9‐knockout mice.^8^ The specific anti‐inflammatory effect has been demonstrated for gCTRP9, which is mediated by the reduction of nuclear factor‐κB (NF‐ƙB) phosphorylation and nuclear translocation (Figure 3b).^21^

### 3.5 Inversion of mitochondrial dysfunction by CTRP9 in cardiomyocytes under IRI

To neutralize the myocytes’ lactic acidosis, accumulated protons and increase nicotinamide adenine dinucleotide (NAD^+^) conversion into NADH, Na^+^/H^+^ pomp is actively exchanged by the intracellular protons with Na^+^ following ischaemia.^36^ At the next step, Na^+^/Ca^2+^ ion‐exchange channels facilitate Na^+^ efflux and intracellular traffic of Ca^2+^, contributing to the elevation of ionized calcium.^36^ The physiological significance of cytosolic Ca^2+^ is highlighted during the reperfusion, where accumulated extracellular H^+^ ions are eliminated. On this basis, the H^+^ gradient occurs across the cell membrane.^26^ Continuous trafficking of Ca^2+^ into the cytosol and accumulation of Ca^2+^ in mitochondrial matrix leads to the impairment of mitochondrial permeability transition pore (mPTP) and simultaneous cell death.^26^ These maladaptive conditions result in moderate to excessive production of ROS and mitochondrial fission.^37^ In this respect, molecular and ultrastructural analyses have indicated that mitophagy is a selective degradation of dysfunctional mitochondria through the autophagy process under various stress conditions that warranties mitochondrial quality control.^37^ It is also noteworthy to mention that several studies have demonstrated a protective role of mitophagy in the setting of ischaemia and IRI.^38^ Because of its unique structure, CTRP9 acts as an adaptor molecule and connects various mediators related to mitophagy.^39^ Relevant to the protective effects of mitophagy in cell resistance, CTRP9 is involved in mitophagy‐related signalling pathways following IRI. CTRP9 contribution in mitophagy leads to the activation of BCL2L13 and subsequent mitophagy flux.^40^ The possible role of CTRP9 in mitophagy flux has also been investigated in terms of lysosomal activity, showing the stimulatory effect of CTRP9 on Cathepsin B and L function after myocardial IRI (Figure 3c).^41^ The activation of mitophagy in cardiomyocytes after IRI implies the early‐stage activity of CTRP9, allowing the host cells to eliminate injured organelles via direction to the lysosomal system; however, the precise underlying mechanism for the activation of mitophagy‐related responses, as well as interaction of CTRP9 with different effectors, are lacking.

## 4 CTRP9 ACTIVITY IN TERMS OF VASCULAR SYSTEM

### 4.1 Atheroprotective role of CTRP9 in cardiomyocytes under IRI

Atherosclerosis is a leading cause of most cardiovascular disorders such as MI. Noteworthy, a complex of signalling networks and various effectors participates in the formation of atherosclerotic lesions. More details associated with the atherosclerosis signalling pathway are highly recommended to develop novel therapeutic approaches for CVD patients.^42^ Different studies have inferred that serum levels of adiponectin and CTRP9 expression were down‐regulated after myocardial IRI and acute MI.^13^, ^32^ These data show that the basal levels of CTRP9 are essential for the maintenance of cardiomyocytes homeostasis and the prevention of several pathologies. Also, the reduction of tissue CTRP9 likely occurs due to highly dynamic biogenesis, depletion of cellular source and shorter lifetime. Whether reducing this factor can be related to compensatory response in IRI needs further investigation. The therapeutic effect of CTRP9 in coronary atherosclerosis is based on the promotion of high‐density lipoprotein cholesterol (HDL‐C) synthesis in patients suffering from diet‐induced obesity.^23^ Besides, another fundamental change driven by CTRP9 is associated with plaque stability mediated by the inhibition of adenosine diphosphate (ADP)‐stimulated platelet activation pathway.^6^, ^9^ CTRP9 can also inhibit cholesterol efflux to the atherosclerotic plaques and the activity of multiplicity of cells and the formation of foam cells associate with the destruction of the vascular lumen. Recent data have provided evidence of transporter activity of CTRP9, being a cholesterol transporter receptor, to promote the cholesterol efflux via increasing ATP‐binding membrane cassette transporter A1 (ABCA1) and G1 (ABCG1) expression level in foam cells.^43^ Given the highly intricate cell connection network within the atherosclerotic plaques, it is mandatory future studies focus on the dynamics of cell population and different cytokines after the activation of CTRP9.

### 4.2 Desirable effects of CTRP9 on angiogenesis

The term angiogenesis (neovascularization) is a complex phenomenon consisting of blood vessel generation from pre‐existing networks.^44^ It is believed that the balance between the pro‐ and anti‐angiogenic factors could dictate a specific endothelial cells (ECs) function, which paves the luminal surface of blood vessels, resulting in vascular growth and/or atresia.^45^ Different works provide data showing the inhibitory effects of CTRP9 on vascular diseases.^46^ As expected, CTRP9 is the potential to suppress endothelial inflammation.^47^ The intracellular level of NF‐kB is elevated at the site of vascular inflammation contributing to EC apoptosis and death. As a consequence of vascular inflammation, the expression of surface endothelial adhesion molecules such as intercellular adhesion molecule‐1 (ICAM‐1) and vascular cell adhesion molecule‐1 (VCAM‐1) is promoted. This alteration is concurrent with accelerated immune cell recruitment and attachment of inflamed cells.^48^ Jung and his colleagues have also confirmed the protective effect of CTRP9 on atherosclerotic human aortic ECs indicated by reduced VCAM‐1 and ICAM‐1 through the AMPK/NF‐kB axis.^47^ Considering pleiotropic effects of AMPK, the modulation of AMPK by CTRP9 can yield to diverse ECs bioactivities. For instance, it was well‐established that the treatment of ECs with CTRP9 could exert endothelium‐dependent vasodilation via the activation of the AMPK/endothelial nitric oxide synthase (eNOS) pathway (Figure 1).^11^ A crucial role of eNOS has also been shown in patients with blood pressure and metabolic disorders.^11^ An example was provided by Yamaguchi et al. They revealed that CTRP9‐knockout mice failed to retrieve the blood flow, as well as decreased capillary density, in the ischaemic limb following unilateral hind limb ischaemic surgery. While the application of an adenoviral vector expressing CTRP9 (Ad‐CTRP9) could accelerate neovascularization and increase microvascular density.^49^

As such, the treatment of human umbilical vein endothelial cells (HUVECs) with recombinant CTRP9 protein significantly enhances cell migration and tubulogenesis after phosphorylation of AMPK, Akt, and eNOS.^49^ Meanwhile, the activation of PI3K/Akt can inhibit the progression of apoptotic changes.^50^ Therefore, AMPK is one of the significant effectors inside the ECs activated by CTRP9 after ischaemia.^13^ It implies that CTRP9 can regulate the activity of AMPK, Akt and p42/44 mitogen‐activated protein kinase (MAPK), as well as reduction of microangiopathies, and bulk vascular diseases during the progression of diabetes.^49^ The existence of EC‐to‐EC tight junction provides a natural vascular barrier, limiting the transfer of biomolecules between the blood and specific tissues.^51^ The breakdown of the blood‐retinal barrier is commonly described in diabetic patients. It has been notified that CTRP9 can diminish retinal vessel inflammation by reducing the levels of IL‐1β, TNF‐α and MCP‐1.^52^ In response to diabetic conditions, serum levels of CTRP9 and total adiponectin concentrations were increased in diabetic patients.^53^ This effect would be related to atherosclerotic plaque formation and abnormal glucose metabolism.^54^ Similar to diabetic conditions, the elevation of CTRP9 during pulmonary arterial hypertension can ameliorate progressive EC injury by induction of eNOS, suppression of both endothelin‐1 (ET‐1) and matrix metalloproteinase‐2 (MMP‐2), and ultimately can reduce the population of apoptotic ECs.^50^ The activation of this factor under different pathological conditions is estimated to correlate with the pleiotropic activity of CTRP9. Whether CTRP9 is involved in innate or specific cell injury is the subject of debate. Therefore, the mutual crosstalk of CTRP9 with different effectors in ECs should be further elucidated either in in vitro or in vivo settings.

### 4.3 Post‐IRI cardioprotective role of CTRP9

The physiological significance of CTRP9 is determined in the heart when the different data tell us about the serum and tissue concentrations. Notably, it has been shown that cardiac CTRP9 level is nearly 1.6‐fold more than that of plasma levels, and a robust reduction occurs in serum levels of CTRP9 following the myocardial IRI.^6^, ^55^ The apparent lack of cardiac CTRP9 activity may be explained by left ventricular (LV) dysfunction in hypertrophic hearts after both severe aortic stenosis and transverse aortic constriction‐induced heart failure.^6^ The ablation of CTRP9 can increase infarct size, lower left ventricular ejection fraction (LVEF) and distended LV end‐systolic diameter following MI, and IRI.^30^ As discussed earlier, the activation of the cardiac AdipoR1‐dependent pathway by CTRP9 elicits cardiovascular functions.^8^

Of note, the cellular distribution of cardiac AdipoR1 remains unaffected during both pathological and physiological conditions.^30^ However, pharmacological inhibition of AdipoR1 does not alter CTRP9 induced anti‐apoptotic properties following MI/IRI.^30^ By contrast, the activation of AdipoR1 facilitates CTRP9‐induced AMPK activation in the cardiac tissue and exogenous CTRP9 administration had no protective effect on cardiac tissue injury in AdipoR1^−/−^ mice.^8^, ^11^, ^12^ These data show that basal levels of AdipoR1 can promote CTRP9 effects under inflammatory conditions. At the same time, selective inhibition of this receptor may provoke alternative membrane‐bound receptors along with the AdipoR1 signalling pathway. Whether receptor‐independent mechanisms are involved in the bioactivity of CTRP9 needs more experiments.

A great body of evidence has shown numerous intracellular effectors for CTRP9. Like ECs, the activation of the AMPK signalling pathway and increased cAMP content have been shown in cultured cardiomyocytes and vascular smooth muscle cells exposed to CTRP9. Importantly, selective inhibition of AMPK by compound C in cardiomyocytes leads to NF‐kB phosphorylation after treatment with LPS, triggering biochemical reactions by increasing phosphorylated AMPK, whereas these effects were blunted in CTRP9^−/−^ mice.^8^ The lack of CTRP9 in cardiomyocytes underlies cellular susceptibility after being exposed to LPS because of the promotion of pro‐inflammatory response. Besides, it is suggested that AMPK is one of the foremost downstream effectors of CTRP9 and its activity depends on CTRP9 levels.

Molecular investigations have also indicated that cells can respond to CTRP9 using multiple intracellular effectors in different ways. For instance, GRP78 is an ERS landmark and elevates simultaneously with the activation of caspase‐12 following MI/IRI.^56^ Therefore, it should be noted that the overexpression of cardiac CTRP9 was indicated to efficiently suppress GRP78 and caspase‐12 and reduce apoptotic death in cardiomyocytes.^57^, ^58^ Although investigated to some extent, the activation of CTRP9 can regulate the function of CRT in ER,^57^, ^58^ the elimination of misfolded proteins and lysosomal degradation reduce the possibility of apoptotic cardiomyocyte death. Based on mounting evidence, it has been demonstrated that CRT can display several functions according to subcellular location. Cell‐membrane bond CRT provides binding sites in the complement C1q, leading to phagocytosis of apoptotic cells.^59^ Also, this receptor can internalize autocrine cardiac CTRP9 following MI/IRI. Similar to CTRP9 effects, the inhibition of CRT in animal models induces apoptotic changes via alteration of the Bcl‐2/Bax ratio and caspase‐12 activity.^60^ The direct interaction of CTRP9 with CRT causes the intracellular Ca^2+^ influx and phosphorylates PKA and cAMP‐response element‐binding protein (CREB).^60^ CREB is a stimulus‐induced transcription factor and triggers the transcription of several pro‐survival target genes in response to different external stimuli like Ca^2+^ influx.^61^ Together, CTRP9–CRT interaction by inhibition of ERS‐related apoptosis and activation of the PKA/CREB signalling pathway can directly protect against the myocardial IRI. This effect is mediated by PKA/CREB activation and is sustained by induction of Bcl‐2 and Bcl‐XL.^30^

## 5 THE THERAPEUTIC EFFECT OF CTRP9 MEDIATED BY AUTOPHAGIC RESPONSE IN IRI

Autophagy (macroautophagy) is considered a conserved catabolic process responsible for cell haemostasis. The autophagy machinery balances cell metabolism by facilitating bulk degradation and recycling of long‑lived proteins or dysfunctional cytosolic organelles.^62^, ^63^ The favourable effects of autophagy on cardiac tissue have been documented in various studies using different models of myocardial IRI.^64^ Evidence for the participation of autophagy‐related signalling pathways has been obtained based on in vitro [glucose deprivation and hypoxia of neonatal murine cardiomyocytes] and in vivo models of coronary artery occlusion.^65^ Recent data have shown that certain glycoproteins activity like Follistatin‐Like 1 (FSTL1) can restore the function of cardiomyocytes following IRI mainly by autophagy.^64^ It was recently shown p27^Kip1^ (p27), a key regulator of tumour suppression, can remarkably increase autophagy response to maintain dying cardiomyocytes exposed to glucose deprivation.^66^ The activation of autophagy at early stages can retrieve cellular function but persistent and overactivation of autophagy leads to cell death and atresia. This discrepancy might be correlated with the intensity and time‐lapse activation of different effectors, which have been shown during MI.^15^ Transcriptomic and proteomic analyses also showed that the activation of specific signalling pathways accompanies desirable effects of autophagy in IRI.to better words, ischaemia stimulates autophagy activity through an AMPK‐dependent manner (AMPK‐mTOR‐ULK1 axis) at the early stages whereas this response switches to Beclin‐1/PI3K‐dependent manner (AKT/Bcl‐2/Beclin‐1 pathway) after prolonged IRI (Figure 3c). Unlike the former pathway, the cardiomyocyte toxicity and death are likely in the latter scenario.^15^, ^67^, ^68^

Along with ROS generation, persistent activation of autophagy can promote cell death following re‐oxygenation mainly due to dysregulated autophagic flux and subsequent autophagosome accumulation.^69^ However, detailed molecular mechanisms supporting the detrimental effect of autophagy on cardiomyocytes should be addressed by further studies. A pro‐oxidant molecule known as thioredoxin‐interacting protein (TXNIP) and an autophagy regulator Redd1 are novel effectors involved in autophagic injury after IRI.^70^ In contrast, studies conducted by different research groups showed therapeutic effects of anti‐thrombin III and trimetazidine following IRI mediated by autophagy suppression through the PI3K/Akt and AKT/mTOR axes, respectively.^71^ Noteworthy, simultaneous activation of glycogen synthase kinase 3b (GSK3b) and extracellular signal‐regulated kinases (ERK1/2) pathways, as major autophagy regulators, enhances cell resistance following IRI in the steatotic liver.^72^ Like hepatic tissue, the interruption of the autophagy signalling pathway exacerbates IRI in kidneys.^73^ Balancing the signals and maintaining the right level of autophagy can be considered as a promising therapeutic strategy for the alleviation of IRI.

### 5.1 Atheroprotective role of CTRP9 mediated by autophagy in favour of IRI treatment

As mentioned above, the AMPK/ mTOR axis constitutes a central route for autophagy flux.^74^ Concerning obtained data, it seems that CTRP9 can also affect autophagy machinery by activating the AMPK/mTOR signalling pathway, particularly under the atherosclerotic condition.^43^ The effect of CTRP9 on autophagy can be speculated from what happens to AMPK. CTRP9 promotes the phosphorylation of AMPK in foam cells (macrophages), in which numerous downstream signalling effectors, mainly mTOR, are inhibited while the protein levels of p62 and LC3‐II (light chain associated protein 3‐II) are retrieved. The activation of autophagic response in foam cells facilitates cholesterol efflux and the expression of ABCA1 and ABCG1.^43^ Enhanced cholesteryl ester hydrolysis and lipid droplet degradation are reported in foam cells with autophagy activity. It has also been shown that CTRP9 significantly up‐regulates autophagy flux in the foam cells.^75^ Therefore, CTRP9 acts as an anti‐atherosclerotic agent via direct efflux of cholesterol and enhancing autophagy activation through the AMPK signalling pathway. Because of the activation of the AMPK/mTOR signalling pathway and increase of palmitic acid efflux in THP‐1 macrophages and HUVECs after treatment with CTRP9,^43^, ^76^, ^77^ one could hypothesize there is a logical relationship between the CTRP9 and autophagy. Despite the fact that recent findings demonstrate a rational interplay between the CTRP9 mechanism of action and autophagy activity, the precise molecular mechanisms remain to elucidate.

## 6 THE PROGNOSTIC POTENTIAL OF CTRP9 REGARDING METABOLIC DISORDERS INDUCED BY DIABETES

As mentioned earlier, CTRP9 has the potential to be an option for improving glucose‐related metabolic syndrome.^78^ Previous data showed that serum levels of leptin and adiponectin were significantly lower in patients with type 2 diabetes,^79^ which would be predictive of circulating CTRP9.^54^, ^79^, ^80^ It has also been indicated that serum levels of CTRP9 were remarkably correlated with systolic pressure and C‐reactive protein levels in diabetic subjects.^80^ In addition, circulating CTRP9 is positively associated with obesity markers and insulin resistance, including body mass index (BMI), fasting blood glucose (FBS) level, insulin and LDL‐C.^81^

Besides, the results of a clinical study demonstrated that serum levels of CTRP9 were significantly diminished in diabetic patients with pulmonary infection^82^ and the first trimester of pregnancy among women with gestational diabetes.^83^, ^84^ Notably, CTRP9 exerted a protective role against diabetic nephropathy, as well as kidney fibrosis in vivo by inhibiting glomerular and tubular glycogen accumulation, apoptosis, and hyperglycaemia‐mediated oxidative stress.^85^ The results of an experimental study also revealed that CTRP9 could augment cell viability and reduce high glucose‐induced oxidative stress and apoptosis via AMPK/Nuclear factor erythroid‐derived 2‐like 2 (NFE2L2) signalling in ARPE‐19 cells, a retinal pigment epithelial (RPE) cell line, indicating CTRP9 as a promising therapeutic target in diabetic retinopathy.^86^ Similarly, a recent evidence‐based study in Egypt also found a negative correlation between serum CTRP9 levels and diabetic retinopathy progression.^87^

Given the clinical significance of circulating CTRP9 for impeding the platelet aggregation, either *CTRP9* gene up‐regulation or administration of exogenous CTRP9 can exhibit a protective effect in diabetic patients who are at high risk for cardiac events like IRI.^88^ Moreover, the serum CTRP9 levels were assessed in paediatrics with type 1 diabetes (T1D) or type 2 (T2D) during a recent cross‐sectional study.^89^ Regression analysis uncovered that the CTRP9 was positively associated with C‐peptide (*p* = 0.006) in T1D compared to T2D patients. Inconsistent with this finding, CTRP9 extensively participates in metabolic homeostasis, especially in paediatric diabetes.^89^ Given that the cardiac expression of CTRP9 has been robustly suppressed under diabetic conditions in vivo,^90^ the administration of exogenous CTRP9 represented a significant structural and functional improvement in the heart tissue. In line with this, overexpression of CTRP9 also exhibited a potent cardioprotective role against IRI in vitro.^90^ Recent clinical advances regarding the therapeutic impacts of CTRP9 in patients with CVD and metabolic disorders have been well‐illustrated in Table 1.

**TABLE 1:** List of clinical studies regarding CTRP9 impacts on CVD and metabolic syndrome co‐morbidities

Author	Year	Patients	Control	Sample	Expression	Mechanism	Outcome
Du et al.^91^	2019	CAD (*N* = 131)	Healthy Control (*N* = 131)	Serum	Down‐regulation	_	CTRP9 may be a novel therapeutic target against pathologic remodeling
Gao et al.^92^	2019	HFrEF (*N* = 168)	Healthy Control (*N* = 176)	Plasma	Down‐regulation	_	CTRP9 are decreased in patients with HfrEF
Moradi et al.^93^	2018	CAD (*N* = 157) T2DM (*N* = 37) CAD+T2DM (*N* = 63)	Symptomatic Non‐CAD Control (*N* = 80)	Serum	Up‐regulation	_	CTRP9 levels were independently associated with increased risk of CAD and T2DM
Appari et al.^6^	2017	Cardiac hypertrophy due to arterial hypertension	Healthy Control	Myocardial Samples	Up‐regulation	ERK Activation	CTRP9 promoted hypertension‐induced cardiac hypertrophy
Hasegawa et al.^94^	2017	A training group (middle‐aged and older participants, *N* = 26)	Sedentary Control Group (Middle‐Aged And Older Participants, *N* = 26)	Serum	No Significant Difference	_	there was no significant difference in the change in serum CTRP9 concentration between the training and control groups
Sara F. Ahmed^95^	2018	Postmenopausal females (*N* = 86), CAD (*N* = 29) T2DM (*N* = 29) CAD+T2DM (*N* = 15)	Healthy Control (*N* = 13)	Serum	Decreased	_	CTRP3 and CTRP9 could be potential markers recommended for the clinical use in the diagnosis, prognosis and follow up of patients with T2D at risk of developing CAD.
Wang et al.^96^	2015	CAD (*N* = 214)	Non‐CAD (*N* = 121)	Serum	Down‐regulation	_	circulating and coronary CTRP9 plays an important role in the inflammation and coronary atherosclerosis Of CAD patients.

Abbreviations: CAD, Coronary artery disease; Hfref, heart failure with reduced ejection fraction; T2DM, type 2 diabetes mellitus.

## 7 CONCLUSION

Advancement in current knowledge about CTRP9 has specified this factor's critical role in protecting cardiomyocytes in different cardiac pathologies. However, precise related molecular mechanisms have not been fully addressed yet. Future experiments should be oriented towards elucidating the pleiotropic effects of CTRP9 in different experimental models of cardiac diseases to derive the translation of basic science to the clinical setting.

## AUTHOR CONTRIBUTIONS

**Seyyed‐Reza Sadat‐Ebrahimi:** Writing – original draft (equal); Writing – review & editing (equal). **Hassan Amini:** Writing – original draft (equal). **Reza Rahbarghazi:** Writing – original draft (equal); Writing – review & editing (equal). **Paria Habibollahi:** Writing – review & editing (equal). **Shahrouz Ghaderi:** Software (lead). **Hadi Rajabi:** Software (equal). **Aysa Rezabakhsh:** Conceptualization (lead); Project administration (equal); Validation (equal); Writing – original draft (equal); Writing – review & editing (equal).

## CONFLICT OF INTEREST

None declared.

## Data Availability

This paper is exempt from data sharing.
